# Does Purple Reign? PCR versus grams stain for critical result reporting for blood cultures growing gram positive cocci

**DOI:** 10.1017/ash.2025.255

**Published:** 2025-09-24

**Authors:** Julia Fischer, Gabryjela Walsh, Humberto Urruita, Adina Paley, Michael Lorenzo, Jennifer Schimmel

**Affiliations:** 1 Baystate Medical Center; 2 Baystate Health

## Abstract

**Background:** Gram positive cocci (GPC) in blood cultures (BLCX) can represent pathogens or contaminants. Many laboratories notify care teams of a positive BLCX with the gram stain (GS) as a critical results report (CRR). However, PCR results are available about 90 minutes later and can provide useful information to distinguish a contaminant from a pathogen. This study aimed to investigate the effects of changing CRR from Gram Strain Results (GSR) to PCR Results (PCRR) on anti-MRSA coverage (AMC) and other healthcare utilization for GPC. **Methods:** Retrospective observational study of adult patients with BLCX growing GPC. Clinical and healthcare utilization information was collected using the electronic medical record. A “true pathogen” (TP) was defined as: MRSA, MSSA, Enterococcus, Streptococcus pyogenes, Streptococcus agalactiae and Streptococcus pneumoniae. We also defined TP as a coagulase-negative staphylococci or other Streptococcus species with 2/2 positive BLCX with intraarticular or endovascular hardware present and modified Pitt score (mPitt) greater than or equal to 4. A “likely contaminant” (LC) was defined as coagulase negative staphylococci or Streptococcus species (not included in the initial TP definition) with 1-2 BLCX positive, with or without intraarticular or endovascular hardware present, mPitt < 4. CRR protocol was changed from a call from the laboratory to the floor upon GSR to a call with the PCRR to relay both the GS and the PCR data. **Results:** Of 167 patients included, 91 had CRR with GSR and 76 had CRR with PCRR. For GSR, 56 were classified as TP and 25 were classified as LC. For PCRR 38 were classified as TP and 37 were classified as LC. Overall, there was more use of AMC for patients with GSR (63%) compared to PCRR (42%) p < 0.05. There was a significant difference in AMC for TP after PCRR (42%) compared to GSR (74%) p < 0 .05. There was no significant difference AMC for LC in PCRR (41%) from GSR (56%) p = 0.37. For LC, there was a decrease in echocardiograms 21% compared to 28% and ID consults 24% compared to 60% respectively with PCRR compared to GSR. **Conclusion:** PCR CRR decreased AMC for TP and for total patients after PCR CRR indicating that changing CRR to PCR may be an important antimicrobial stewardship tool. For LC, there was a significant decrease in ID consults and echocardiograms after changing to PCR for CRR indicating PCR CRR may be an important tool for healthcare resource utilization.

